# Supplementary material for: Content and form of original research articles in general major medical journals
Source: PLoS One. 2023 Jun 28;18(6):e0287677. doi: 10.1371/journal.pone.0287677 (PMC10306213; doi:10.1371/journal.pone.0287677)
Supplement: S3 File — (HTML) [file pone.0287677.s003.html]

Title content and form of original research articles in high-ranked medical journals


# Title content and form of original research articles in high-ranked medical journals

### Title content and form of research articles

#### PLOS ONE

#### Nicole Heßlera & Andreas Zieglerb,c,d,\*

#### aInstitut für Medizinische Biometrie und Statistik (IMBS), Universität zu Lübeck, Universitätsklinikum-Schleswig-Holstein, Campus Lübeck, Lübeck, Germany

#### bMedizincampus Davos, Davos, Switzerland

#### cSchool of Mathematics, Statistics and Computer Science; University of KwaZulu Natal, Pietermaritzburg, South Africa

#### dDepartment of Cardiology, University Heart & Vascular Center Hamburg, University Medical Center Hamburg-Eppendorf, Hamburg, Germany

#### Correspondence:\* ziegler.lit@mailbox.org

## Table of Contents

- 1
  Abbreviations
- 2
  Notes
- 3 Descriptive statistics
  - 3.1
    Content
  - 3.2
    Form
- 4 Meta-analyses - Content
  - 4.1 Topic - Methods
    - 4.1.1 Methods mention
      - 4.1.1.1 Per journal
      - 4.1.1.2
        Per year
      - 4.1.1.3 Per journal and year
    - 4.1.2 Patient population
      - 4.1.2.1 Per journal
      - 4.1.2.2 Per year
      - 4.1.2.3 Per journal and year
    - 4.1.3
      Geography
      - 4.1.3.1 Per journal
      - 4.1.3.2 Per year
      - 4.1.3.3 Per journal and year
    - 4.1.4 Clincial context
      - 4.1.4.1 Per journal
      - 4.1.4.2 Per year
      - 4.1.4.3 Per journal and year
    - 4.1.5
      Treatment
      - 4.1.5.1 Per journal
      - 4.1.5.2 Per year
      - 4.1.5.3 Per journal and year
    - 4.1.6
      Study name
      - 4.1.6.1 Per journal
      - 4.1.6.2 Per year
      - 4.1.6.3 Per journal and year
  - 4.2 Topic - Results
    - 4.2.1 Results mention
    - 4.2.2 Quantitative
      information
    - 4.2.3 Semi-quantitative
      information
    - 4.2.4
      Relation
      - 4.2.4.1 Per journal
      - 4.2.4.2 Per year
      - 4.2.4.3 Per journal and year
- 5 Meta-analyses - Form
  - 5.1 Topic - Methods
    - 5.1.1 Abbreviation
      - 5.1.1.1 Per journal
      - 5.1.1.2 Per year
      - 5.1.1.3 Per journal and year
    - 5.1.2
      Dash
    - 5.1.3
      Subtitle
      - 5.1.3.1 Per journal
      - 5.1.3.2 Per year
      - 5.1.3.3 Per journal and year
  - 5.2 Topic - Conclusion/Discussion
    - 5.2.1 Declarative titles
    - 5.2.2 Phrasal titles
      - 5.2.2.1 Per journal
      - 5.2.2.2 Per year
      - 5.2.2.3 Per journal and year
    - 5.2.3
      Question
- 6 Logistic regression models -
  Content
  - 6.1 Topic - Methods
    - 6.1.1 Methods mention
    - 6.1.2 Patient population
    - 6.1.3
      Geography
    - 6.1.4 Clinical context
    - 6.1.5
      Treatment
    - 6.1.6 Study name
  - 6.2 Topic - Results
    - 6.2.1 Results mention
    - 6.2.2 Quantitative
      information
    - 6.2.3 Semi-quantitative
      information
    - 6.2.4
      Relation
- 7 Logistic regression models -
  Form
  - 7.1 Topic - Methods
    - 7.1.1 Abbreviation
    - 7.1.2
      Dash
    - 7.1.3
      Subtitle
  - 7.2 Topic - Conclusion/Discussion
    - 7.2.1 Declarative titles
    - 7.2.2 Phrasal titles
    - 7.2.3
      Question
- 8 Comparison with Kerans et al. 
  - 8.1 Methods mention
  - 8.2 Patient population
  - 8.3
    Geography
  - 8.4 Clinical context
  - 8.5
    Treatment

# 1 Abbreviations

BMJ = The BMJ  
CI = confidence interval  
JAMA = Journal of the American Medical Association  
New Engl J Med = New England Journal of Medicine  
OR = odds ratio  
PLoS Med = PLoS Medicine  
RE = random effect  
SE = standard error

# 2 Notes

**Meta-analyses**: The logit transformation was used for
the meta-analysis of binary variables for estimating the pooled
proportions, standard errors were not back transformed. Analyses were
only performed, if appropriate.

**Regression models**: For all regression models, BMJ
was used as reference category. Time and journal were used as
covariables. Analyses were only performed, if appropriate.

# 3 Descriptive statistics

From each journal, ten original articles per year, i.e. 100 articles
per journal in total, were randomly chosen for the assessments of
defined items from two independent raters; see manuscript for detailed
description of items.

## 3.1 Content

| **Characteristic** | **BMJ**, N = 1001 | **JAMA**, N = 1001 | **Lancet**, N = 1001 | **New Engl J Med**, N = 1001 | **PLoS Med**, N = 1001 |
| --- | --- | --- | --- | --- | --- |
| Methods mention |  |  |  |  |  |
| 0 | 2 (2%) | 53 (53%) | 7 (7%) | 89 (89%) | 4 (4%) |
| 1 | 98 (98%) | 47 (47%) | 93 (93%) | 11 (11%) | 95 (96%) |
| Unclear | 0 | 0 | 0 | 0 | 1 |
| Patient population |  |  |  |  |  |
| 0 | 38 (38%) | 24 (24%) | 28 (28%) | 22 (22%) | 30 (30%) |
| 1 | 62 (62%) | 76 (76%) | 72 (72%) | 78 (78%) | 70 (70%) |
| Geography |  |  |  |  |  |
| 0 | 69 (69%) | 84 (84%) | 83 (83%) | 91 (91%) | 48 (48%) |
| 1 | 31 (31%) | 16 (16%) | 17 (17%) | 9 (9%) | 52 (52%) |
| Clincial context |  |  |  |  |  |
| 0 | 27 (27%) | 19 (19%) | 17 (17%) | 11 (11%) | 19 (19%) |
| 1 | 73 (73%) | 81 (81%) | 83 (83%) | 89 (89%) | 81 (81%) |
| Treatment |  |  |  |  |  |
| 0 | 70 (70%) | 45 (45%) | 38 (38%) | 42 (42%) | 73 (73%) |
| 1 | 30 (30%) | 55 (55%) | 62 (62%) | 58 (58%) | 27 (27%) |
| Study name |  |  |  |  |  |
| 0 | 89 (89%) | 80 (80%) | 55 (55%) | 100 (100%) | 90 (90%) |
| 1 | 11 (11%) | 20 (20%) | 45 (45%) | 0 (0%) | 10 (10%) |
| Results mention |  |  |  |  |  |
| 0 | 99 (99%) | 99 (100%) | 99 (99%) | 97 (98%) | 98 (98%) |
| 1 | 1 (1%) | 0 (0%) | 1 (1%) | 2 (2%) | 2 (2%) |
| Unclear | 0 | 1 | 0 | 1 | 0 |
| Quantitative information |  |  |  |  |  |
| 0 | 100 (100%) | 100 (100%) | 100 (100%) | 100 (100%) | 100 (100%) |
| Semi-quantitative information |  |  |  |  |  |
| 0 | 100 (100%) | 100 (100%) | 99 (99%) | 98 (98%) | 99 (99%) |
| 1 | 0 (0%) | 0 (0%) | 1 (1%) | 2 (2%) | 1 (1%) |
| Relation |  |  |  |  |  |
| 0 | 48 (48%) | 32 (32%) | 65 (65%) | 77 (77%) | 43 (43%) |
| 1 | 52 (52%) | 68 (68%) | 35 (35%) | 23 (23%) | 57 (57%) |
|  |  |  |  |  |  |
| --- | --- | --- | --- | --- | --- |
| 1 n (%) | | | | | |

## 3.2 Form

| **Characteristic** | **BMJ**, N = 1001 | **JAMA**, N = 1001 | **Lancet**, N = 1001 | **New Engl J Med**, N = 1001 | **PLoS Med**, N = 1001 |
| --- | --- | --- | --- | --- | --- |
| Abbreviation |  |  |  |  |  |
| 0 | 69 (69%) | 56 (56%) | 45 (45%) | 76 (76%) | 68 (68%) |
| 1 | 31 (31%) | 44 (44%) | 55 (55%) | 24 (24%) | 32 (32%) |
| Dash |  |  |  |  |  |
| 0 | 97 (97%) | 100 (100%) | 100 (100%) | 98 (98%) | 100 (100%) |
| 1 | 3 (3%) | 0 (0%) | 0 (0%) | 2 (2%) | 0 (0%) |
| Subtitle |  |  |  |  |  |
| 0 | 1 (1%) | 59 (59%) | 1 (1%) | 98 (98%) | 2 (2%) |
| 1 | 99 (99%) | 41 (41%) | 99 (99%) | 2 (2%) | 98 (98%) |
| Declarative title |  |  |  |  |  |
| 0 | 100 (100%) | 100 (100%) | 100 (100%) | 100 (100%) | 100 (100%) |
| Phrasal title |  |  |  |  |  |
| 0 | 89 (89%) | 97 (97%) | 88 (88%) | 93 (93%) | 85 (85%) |
| 1 | 11 (11%) | 3 (3%) | 12 (12%) | 7 (7%) | 15 (15%) |
| Question |  |  |  |  |  |
| 0 | 99 (99%) | 100 (100%) | 99 (99%) | 100 (100%) | 99 (99%) |
| 1 | 1 (1%) | 0 (0%) | 1 (1%) | 0 (0%) | 1 (1%) |
|  |  |  |  |  |  |
| --- | --- | --- | --- | --- | --- |
| 1 n (%) | | | | | |

# 4 Meta-analyses - Content

## 4.1 Topic - Methods

### 4.1.1 Methods mention

#### 4.1.1.1 Per journal

Random effect meta-analyses per journal - methods mention
(logit transformed proportion)

|  | Estimate | SE | Lower 95% CI | Upper 95% CI |
| --- | --- | --- | --- | --- |
| BMJ | 0.94 | 0.42 | 0.88 | 0.97 |
| JAMA | 0.49 | 0.21 | 0.39 | 0.59 |
| Lancet | 0.89 | 0.35 | 0.81 | 0.94 |
| New Engl J Med | 0.13 | 0.31 | 0.08 | 0.22 |
| PLoS Med | 0.92 | 0.39 | 0.84 | 0.96 |

#### 4.1.1.2 Per year

Random effect meta-analyses per year - methods mention (logit
transformed proportion)

|  | Estimate | SE | Lower 95% CI | Upper 95% CI |
| --- | --- | --- | --- | --- |
| 2011 | 0.64 | 1.31 | 0.12 | 0.96 |
| 2012 | 0.70 | 0.77 | 0.34 | 0.91 |
| 2013 | 0.71 | 1.04 | 0.24 | 0.95 |
| 2014 | 0.70 | 0.77 | 0.35 | 0.91 |
| 2015 | 0.71 | 0.84 | 0.32 | 0.93 |
| 2016 | 0.74 | 0.95 | 0.30 | 0.95 |
| 2017 | 0.78 | 1.01 | 0.33 | 0.96 |
| 2018 | 0.69 | 0.81 | 0.32 | 0.92 |
| 2019 | 0.75 | 0.93 | 0.33 | 0.95 |
| 2020 | 0.78 | 1.01 | 0.33 | 0.96 |

#### 4.1.1.3 Per journal and year

Random effect meta-analyses per journal and year - methods
mention (logit transformed proportion)

|  | Estimate | SE | Lower 95% CI | Upper 95% CI |
| --- | --- | --- | --- | --- |
| Overall | 0.72 | 0.26 | 0.6 | 0.81 |

### 4.1.2 Patient population

#### 4.1.2.1 Per journal

Random effect meta-analyses per journal - patient population
(logit transformed proportion)

|  | Estimate | SE | Lower 95% CI | Upper 95% CI |
| --- | --- | --- | --- | --- |
| BMJ | 0.61 | 0.21 | 0.51 | 0.71 |
| JAMA | 0.74 | 0.24 | 0.64 | 0.82 |
| Lancet | 0.70 | 0.23 | 0.60 | 0.79 |
| New Engl J Med | 0.75 | 0.25 | 0.65 | 0.83 |
| PLoS Med | 0.67 | 0.28 | 0.54 | 0.78 |

#### 4.1.2.2 Per year

Random effect meta-analyses per year - patient population
(logit transformed proportion)

|  | Estimate | SE | Lower 95% CI | Upper 95% CI |
| --- | --- | --- | --- | --- |
| 2011 | 0.63 | 0.31 | 0.49 | 0.76 |
| 2012 | 0.70 | 0.31 | 0.55 | 0.81 |
| 2013 | 0.62 | 0.31 | 0.48 | 0.75 |
| 2014 | 0.73 | 0.33 | 0.59 | 0.84 |
| 2015 | 0.74 | 0.35 | 0.59 | 0.85 |
| 2016 | 0.64 | 0.45 | 0.43 | 0.81 |
| 2017 | 0.68 | 0.37 | 0.50 | 0.81 |
| 2018 | 0.73 | 0.35 | 0.58 | 0.84 |
| 2019 | 0.75 | 0.37 | 0.59 | 0.86 |
| 2020 | 0.74 | 0.34 | 0.60 | 0.85 |

#### 4.1.2.3 Per journal and year

Random effect meta-analyses per journal and year - patient
population (logit transformed proportion)

|  | Estimate | SE | Lower 95% CI | Upper 95% CI |
| --- | --- | --- | --- | --- |
| Overall | 0.69 | 0.1 | 0.65 | 0.73 |

### 4.1.3 Geography

#### 4.1.3.1 Per journal

Random effect meta-analyses per journal - geography (logit
transformed proportion)

|  | Estimate | SE | Lower 95% CI | Upper 95% CI |
| --- | --- | --- | --- | --- |
| BMJ | 0.33 | 0.23 | 0.24 | 0.43 |
| JAMA | 0.19 | 0.27 | 0.12 | 0.29 |
| Lancet | 0.20 | 0.27 | 0.13 | 0.30 |
| New Engl J Med | 0.11 | 0.33 | 0.06 | 0.20 |
| PLoS Med | 0.51 | 0.25 | 0.39 | 0.63 |

#### 4.1.3.2 Per year

Random effect meta-analyses per year - geography (logit
transformed proportion)

|  | Estimate | SE | Lower 95% CI | Upper 95% CI |
| --- | --- | --- | --- | --- |
| 2011 | 0.22 | 0.58 | 0.09 | 0.47 |
| 2012 | 0.21 | 0.36 | 0.12 | 0.35 |
| 2013 | 0.24 | 0.53 | 0.10 | 0.46 |
| 2014 | 0.27 | 0.58 | 0.10 | 0.53 |
| 2015 | 0.20 | 0.39 | 0.10 | 0.35 |
| 2016 | 0.27 | 0.55 | 0.11 | 0.53 |
| 2017 | 0.24 | 0.38 | 0.13 | 0.40 |
| 2018 | 0.36 | 0.33 | 0.23 | 0.52 |
| 2019 | 0.29 | 0.49 | 0.13 | 0.51 |
| 2020 | 0.34 | 0.63 | 0.13 | 0.64 |

#### 4.1.3.3 Per journal and year

Random effect meta-analyses per journal and year - geography
(logit transformed proportion)

|  | Estimate | SE | Lower 95% CI | Upper 95% CI |
| --- | --- | --- | --- | --- |
| Overall | 0.27 | 0.14 | 0.22 | 0.32 |

### 4.1.4 Clincial context

#### 4.1.4.1 Per journal

Random effect meta-analyses per journal - clinical context
(logit transformed proportion)

|  | Estimate | SE | Lower 95% CI | Upper 95% CI |
| --- | --- | --- | --- | --- |
| BMJ | 0.70 | 0.24 | 0.60 | 0.79 |
| JAMA | 0.78 | 0.26 | 0.68 | 0.85 |
| Lancet | 0.79 | 0.27 | 0.69 | 0.87 |
| New Engl J Med | 0.86 | 0.31 | 0.76 | 0.92 |
| PLoS Med | 0.78 | 0.30 | 0.67 | 0.87 |

#### 4.1.4.2 Per year

Random effect meta-analyses per year - clinical context (logit
transformed proportion)

|  | Estimate | SE | Lower 95% CI | Upper 95% CI |
| --- | --- | --- | --- | --- |
| 2011 | 0.70 | 0.33 | 0.55 | 0.81 |
| 2012 | 0.75 | 0.42 | 0.56 | 0.87 |
| 2013 | 0.75 | 0.37 | 0.59 | 0.86 |
| 2014 | 0.88 | 0.44 | 0.75 | 0.94 |
| 2015 | 0.79 | 0.44 | 0.62 | 0.90 |
| 2016 | 0.74 | 0.42 | 0.56 | 0.87 |
| 2017 | 0.84 | 0.40 | 0.70 | 0.92 |
| 2018 | 0.75 | 0.34 | 0.60 | 0.85 |
| 2019 | 0.87 | 0.50 | 0.71 | 0.95 |
| 2020 | 0.83 | 0.41 | 0.68 | 0.91 |

#### 4.1.4.3 Per journal and year

Random effect meta-analyses per journal and year - clincial
context (logit transformed proportion)

|  | Estimate | SE | Lower 95% CI | Upper 95% CI |
| --- | --- | --- | --- | --- |
| Overall | 0.78 | 0.12 | 0.73 | 0.81 |

### 4.1.5 Treatment

#### 4.1.5.1 Per journal

Random effect meta-analyses per journal - treatment (logit
transformed proportion)

|  | Estimate | SE | Lower 95% CI | Upper 95% CI |
| --- | --- | --- | --- | --- |
| BMJ | 0.31 | 0.22 | 0.23 | 0.42 |
| JAMA | 0.57 | 0.21 | 0.46 | 0.67 |
| Lancet | 0.60 | 0.27 | 0.47 | 0.72 |
| New Engl J Med | 0.58 | 0.21 | 0.47 | 0.67 |
| PLoS Med | 0.31 | 0.24 | 0.22 | 0.42 |

#### 4.1.5.2 Per year

Random effect meta-analyses per year - treatment (logit
transformed proportion)

|  | Estimate | SE | Lower 95% CI | Upper 95% CI |
| --- | --- | --- | --- | --- |
| 2011 | 0.43 | 0.45 | 0.24 | 0.65 |
| 2012 | 0.45 | 0.43 | 0.26 | 0.66 |
| 2013 | 0.35 | 0.37 | 0.20 | 0.52 |
| 2014 | 0.46 | 0.29 | 0.33 | 0.60 |
| 2015 | 0.40 | 0.50 | 0.20 | 0.64 |
| 2016 | 0.49 | 0.30 | 0.35 | 0.64 |
| 2017 | 0.51 | 0.36 | 0.34 | 0.68 |
| 2018 | 0.56 | 0.36 | 0.38 | 0.72 |
| 2019 | 0.51 | 0.55 | 0.26 | 0.75 |
| 2020 | 0.55 | 0.61 | 0.27 | 0.80 |

#### 4.1.5.3 Per journal and year

Random effect meta-analyses per journal and year - treatment
(logit transformed proportion)

|  | Estimate | SE | Lower 95% CI | Upper 95% CI |
| --- | --- | --- | --- | --- |
| Overall | 0.47 | 0.12 | 0.41 | 0.53 |

### 4.1.6 Study name

#### 4.1.6.1 Per journal

Random effect meta-analyses per journal - study acronym (logit
transformed proportion)

|  | Estimate | SE | Lower 95% CI | Upper 95% CI |
| --- | --- | --- | --- | --- |
| BMJ | 0.14 | 0.39 | 0.07 | 0.26 |
| JAMA | 0.23 | 0.26 | 0.15 | 0.33 |
| Lancet | 0.46 | 0.24 | 0.35 | 0.58 |
| PLoS Med | 0.13 | 0.32 | 0.07 | 0.22 |

#### 4.1.6.2 Per year

Random effect meta-analyses per year - study acronym (logit
transformed proportion)

|  | Estimate | SE | Lower 95% CI | Upper 95% CI |
| --- | --- | --- | --- | --- |
| 2011 | 0.16 | 1.12 | 0.02 | 0.63 |
| 2012 | 0.19 | 0.44 | 0.09 | 0.36 |
| 2013 | 0.13 | 0.48 | 0.06 | 0.28 |
| 2014 | 0.19 | 0.44 | 0.09 | 0.36 |
| 2015 | 0.21 | 0.65 | 0.07 | 0.49 |
| 2016 | 0.28 | 0.50 | 0.13 | 0.51 |
| 2017 | 0.25 | 0.43 | 0.12 | 0.44 |
| 2018 | 0.19 | 0.62 | 0.06 | 0.43 |
| 2019 | 0.44 | 0.52 | 0.22 | 0.68 |
| 2020 | 0.29 | 0.60 | 0.11 | 0.57 |

#### 4.1.6.3 Per journal and year

Random effect meta-analyses per journal and year - study
acronym (logit transformed proportion)

|  | Estimate | SE | Lower 95% CI | Upper 95% CI |
| --- | --- | --- | --- | --- |
| Overall | 0.2 | 0.17 | 0.15 | 0.26 |

## 4.2 Topic - Results

### 4.2.1 Results mention

Not performed because of low frequencies of articles that mentioned
results in their titles.

### 4.2.2 Quantitative information

Not performed because all articles did not have a quantitative
information in their titles.

### 4.2.3 Semi-quantitative information

Not performed because of low frequencies of articles with
semi-quantitative information in their titles.

### 4.2.4 Relation

#### 4.2.4.1 Per journal

Random effect meta-analyses per journal - relation (logit
transformed proportion)

|  | Estimate | SE | Lower 95% CI | Upper 95% CI |
| --- | --- | --- | --- | --- |
| BMJ | 0.51 | 0.23 | 0.40 | 0.62 |
| JAMA | 0.67 | 0.29 | 0.53 | 0.78 |
| Lancet | 0.36 | 0.21 | 0.27 | 0.46 |
| New Engl J Med | 0.25 | 0.29 | 0.16 | 0.37 |
| PLoS Med | 0.57 | 0.28 | 0.43 | 0.69 |

#### 4.2.4.2 Per year

Random effect meta-analyses per year - relation (logit
transformed proportion)

|  | Estimate | SE | Lower 95% CI | Upper 95% CI |
| --- | --- | --- | --- | --- |
| 2011 | 0.48 | 0.45 | 0.28 | 0.69 |
| 2012 | 0.59 | 0.39 | 0.41 | 0.76 |
| 2013 | 0.31 | 0.31 | 0.19 | 0.45 |
| 2014 | 0.46 | 0.40 | 0.28 | 0.65 |
| 2015 | 0.44 | 0.37 | 0.28 | 0.62 |
| 2016 | 0.40 | 0.38 | 0.24 | 0.59 |
| 2017 | 0.60 | 0.35 | 0.43 | 0.75 |
| 2018 | 0.48 | 0.61 | 0.22 | 0.76 |
| 2019 | 0.59 | 0.48 | 0.36 | 0.79 |
| 2020 | 0.36 | 0.64 | 0.14 | 0.66 |

#### 4.2.4.3 Per journal and year

Random effect meta-analyses per journal and year - relation
(logit transformed proportion)

|  | Estimate | SE | Lower 95% CI | Upper 95% CI |
| --- | --- | --- | --- | --- |
| Overall | 0.47 | 0.13 | 0.41 | 0.54 |

# 5 Meta-analyses - Form

## 5.1 Topic - Methods

### 5.1.1 Abbreviation

#### 5.1.1.1 Per journal

Random effect meta-analyses per journal - abbreviation (logit
transformed proportion)

|  | Estimate | SE | Lower 95% CI | Upper 95% CI |
| --- | --- | --- | --- | --- |
| BMJ | 0.32 | 0.24 | 0.23 | 0.43 |
| JAMA | 0.46 | 0.22 | 0.35 | 0.56 |
| Lancet | 0.53 | 0.27 | 0.40 | 0.66 |
| New Engl J Med | 0.27 | 0.24 | 0.19 | 0.37 |
| PLoS Med | 0.33 | 0.22 | 0.24 | 0.43 |

#### 5.1.1.2 Per year

Random effect meta-analyses per year- abbreviation (logit
transformed proportion)

|  | Estimate | SE | Lower 95% CI | Upper 95% CI |
| --- | --- | --- | --- | --- |
| 2011 | 0.31 | 0.63 | 0.12 | 0.61 |
| 2012 | 0.26 | 0.42 | 0.13 | 0.44 |
| 2013 | 0.30 | 0.31 | 0.19 | 0.45 |
| 2014 | 0.31 | 0.31 | 0.19 | 0.45 |
| 2015 | 0.39 | 0.31 | 0.25 | 0.54 |
| 2016 | 0.37 | 0.33 | 0.24 | 0.53 |
| 2017 | 0.34 | 0.30 | 0.22 | 0.48 |
| 2018 | 0.48 | 0.33 | 0.33 | 0.64 |
| 2019 | 0.63 | 0.30 | 0.48 | 0.76 |
| 2020 | 0.38 | 0.43 | 0.21 | 0.59 |

#### 5.1.1.3 Per journal and year

Random effect meta-analyses per journal and year - abbreviation
(logit transformed proportion)

|  | Estimate | SE | Lower 95% CI | Upper 95% CI |
| --- | --- | --- | --- | --- |
| Overall | 0.38 | 0.11 | 0.33 | 0.43 |

### 5.1.2 Dash

Not performed because of low frequencies of articles that contained a
dash in their titles.

### 5.1.3 Subtitle

#### 5.1.3.1 Per journal

Random effect meta-analyses per journal - subtitle (logit
transformed proportion)

|  | Estimate | SE | Lower 95% CI | Upper 95% CI |
| --- | --- | --- | --- | --- |
| BMJ | 0.95 | 0.44 | 0.88 | 0.98 |
| JAMA | 0.44 | 0.22 | 0.34 | 0.55 |
| Lancet | 0.95 | 0.44 | 0.88 | 0.98 |
| New Engl J Med | 0.06 | 0.42 | 0.03 | 0.12 |
| PLoS Med | 0.94 | 0.42 | 0.88 | 0.97 |

#### 5.1.3.2 Per year

Random effect meta-analyses per year - subtitle (logit
transformed proportion)

|  | Estimate | SE | Lower 95% CI | Upper 95% CI |
| --- | --- | --- | --- | --- |
| 2011 | 0.59 | 1.27 | 0.11 | 0.95 |
| 2012 | 0.74 | 0.95 | 0.30 | 0.95 |
| 2013 | 0.74 | 1.14 | 0.23 | 0.96 |
| 2014 | 0.72 | 1.20 | 0.20 | 0.96 |
| 2015 | 0.69 | 1.11 | 0.20 | 0.95 |
| 2016 | 0.76 | 1.09 | 0.27 | 0.96 |
| 2017 | 0.77 | 1.05 | 0.31 | 0.96 |
| 2018 | 0.72 | 1.20 | 0.20 | 0.96 |
| 2019 | 0.77 | 1.05 | 0.31 | 0.96 |
| 2020 | 0.78 | 1.01 | 0.33 | 0.96 |

#### 5.1.3.3 Per journal and year

Random effect meta-analyses per journal and year - subtitle
(logit transformed proportion)

|  | Estimate | SE | Lower 95% CI | Upper 95% CI |
| --- | --- | --- | --- | --- |
| Overall | 0.73 | 0.3 | 0.6 | 0.83 |

## 5.2 Topic - Conclusion/Discussion

### 5.2.1 Declarative titles

Not performed because all articles considered did not have a
declarative title.

### 5.2.2 Phrasal titles

#### 5.2.2.1 Per journal

Random effect meta-analyses per journal - phrasal title (logit
transformed proportion)

|  | Estimate | SE | Lower 95% CI | Upper 95% CI |
| --- | --- | --- | --- | --- |
| BMJ | 0.14 | 0.31 | 0.08 | 0.24 |
| JAMA | 0.07 | 0.41 | 0.03 | 0.13 |
| Lancet | 0.15 | 0.30 | 0.09 | 0.24 |
| New Engl J Med | 0.10 | 0.35 | 0.05 | 0.18 |
| PLoS Med | 0.17 | 0.28 | 0.11 | 0.27 |

#### 5.2.2.2 Per year

Random effect meta-analyses per year - phrasal title (logit
transformed proportion)

|  | Estimate | SE | Lower 95% CI | Upper 95% CI |
| --- | --- | --- | --- | --- |
| 2011 | 0.17 | 0.42 | 0.08 | 0.32 |
| 2012 | 0.17 | 0.41 | 0.09 | 0.32 |
| 2013 | 0.17 | 0.40 | 0.09 | 0.32 |
| 2014 | 0.11 | 0.48 | 0.05 | 0.24 |
| 2015 | 0.09 | 0.50 | 0.04 | 0.21 |
| 2016 | 0.12 | 0.46 | 0.05 | 0.25 |
| 2017 | 0.08 | 0.52 | 0.03 | 0.20 |
| 2018 | 0.14 | 0.44 | 0.06 | 0.27 |
| 2019 | 0.14 | 0.45 | 0.06 | 0.29 |
| 2020 | 0.06 | 0.60 | 0.02 | 0.17 |

#### 5.2.2.3 Per journal and year

Random effect meta-analyses per journal and year - phrasal
title (logit transformed proportion)

|  | Estimate | SE | Lower 95% CI | Upper 95% CI |
| --- | --- | --- | --- | --- |
| Overall | 0.13 | 0.15 | 0.1 | 0.16 |

### 5.2.3 Question

Not performed because of low frequencies of articles that contained a
“?” in their titles.

# 6 Logistic regression models - Content

## 6.1 Topic - Methods

### 6.1.1 Methods mention

The likelihood ratio test revealed a p-value of <0.001 for the
comparison of the full model (journal and time as covariates) and the
reduced model (only time as covariate).

Logistic regression model - methods mention

|  | OR | Lower 95% CI | Upper 95% CI | p-value |
| --- | --- | --- | --- | --- |
| Time | 1.12 | 1.01 | 1.24 | 0.025 |
| JAMA | 0.02 | 0.01 | 0.08 | < 0.001 |
| Lancet | 0.32 | 0.07 | 1.36 | 0.122 |
| New Engl J Med | 0.00 | 0.00 | 0.01 | < 0.001 |
| PLoS Med | 0.54 | 0.11 | 2.60 | 0.439 |

Logistic regression model with multiple comparisons - methods
mention


|  | OR | Lower 95% CI | Upper 95% CI | Adjusted p-value |
| --- | --- | --- | --- | --- |
| JAMA - BMJ | 0.02 | 0.01 | 0.08 | < 0.001 |
| Lancet - BMJ | 0.32 | 0.07 | 1.36 | 0.514 |
| New Engl J Med - BMJ | 0.00 | 0.00 | 0.01 | < 0.001 |
| PLoS Med - BMJ | 0.54 | 0.11 | 2.60 | 0.934 |
| Lancet - JAMA | 14.62 | 6.24 | 34.26 | < 0.001 |
| New Engl J Med - JAMA | 0.14 | 0.07 | 0.29 | < 0.001 |
| PLoS Med - JAMA | 24.92 | 8.87 | 70.00 | < 0.001 |
| New Engl J Med - Lancet | 0.01 | 0.00 | 0.03 | < 0.001 |
| PLoS Med - Lancet | 1.70 | 0.51 | 5.70 | 0.903 |
| PLoS Med - New Engl J Med | 177.74 | 56.65 | 557.65 | < 0.001 |

### 6.1.2 Patient population

The likelihood ratio test revealed a p-value of 0.11 for the
comparison of the full model (journal and time as covariates) and the
reduced model (only time as covariate).

Logistic regression model - patient population

|  | OR | Lower 95% CI | Upper 95% CI | p-value |
| --- | --- | --- | --- | --- |
| Time | 1.06 | 0.99 | 1.13 | 0.100 |
| JAMA | 1.93 | 1.05 | 3.55 | 0.035 |
| Lancet | 1.57 | 0.87 | 2.85 | 0.138 |
| New Engl J Med | 2.16 | 1.16 | 4.01 | 0.015 |
| PLoS Med | 1.43 | 0.79 | 2.57 | 0.238 |

Logistic regression model with multiple comparisons - patient
population


|  | OR | Lower 95% CI | Upper 95% CI | Adjusted p-value |
| --- | --- | --- | --- | --- |
| JAMA - BMJ | 1.93 | 1.05 | 3.55 | 0.218 |
| Lancet - BMJ | 1.57 | 0.87 | 2.85 | 0.572 |
| New Engl J Med - BMJ | 2.16 | 1.16 | 4.01 | 0.110 |
| PLoS Med - BMJ | 1.43 | 0.79 | 2.57 | 0.762 |
| Lancet - JAMA | 0.81 | 0.43 | 1.53 | 0.969 |
| New Engl J Med - JAMA | 1.12 | 0.58 | 2.16 | 0.997 |
| PLoS Med - JAMA | 0.74 | 0.40 | 1.38 | 0.880 |
| New Engl J Med - Lancet | 1.37 | 0.72 | 2.61 | 0.870 |
| PLoS Med - Lancet | 0.91 | 0.49 | 1.67 | 0.998 |
| PLoS Med - New Engl J Med | 0.66 | 0.35 | 1.25 | 0.709 |

### 6.1.3 Geography

The likelihood ratio test revealed a p-value of <0.001 for the
comparison of the full model (journal and time as covariates) and the
reduced model (only time as covariate).

Logistic regression model - geography

|  | OR | Lower 95% CI | Upper 95% CI | p-value |
| --- | --- | --- | --- | --- |
| Time | 1.07 | 0.99 | 1.16 | 0.072 |
| JAMA | 0.43 | 0.22 | 0.85 | 0.015 |
| Lancet | 0.46 | 0.24 | 0.90 | 0.023 |
| New Engl J Med | 0.23 | 0.10 | 0.50 | < 0.001 |
| PLoS Med | 2.40 | 1.35 | 4.29 | 0.003 |

Logistic regression model with multiple comparisons -
geography


|  | OR | Lower 95% CI | Upper 95% CI | Adjusted p-value |
| --- | --- | --- | --- | --- |
| JAMA - BMJ | 0.43 | 0.22 | 0.85 | 0.103 |
| Lancet - BMJ | 0.46 | 0.24 | 0.90 | 0.153 |
| New Engl J Med - BMJ | 0.23 | 0.10 | 0.50 | 0.002 |
| PLoS Med - BMJ | 2.40 | 1.35 | 4.29 | 0.025 |
| Lancet - JAMA | 1.07 | 0.51 | 2.26 | 1.000 |
| New Engl J Med - JAMA | 0.53 | 0.23 | 1.25 | 0.590 |
| PLoS Med - JAMA | 5.60 | 2.89 | 10.86 | < 0.001 |
| New Engl J Med - Lancet | 0.49 | 0.21 | 1.15 | 0.474 |
| PLoS Med - Lancet | 5.22 | 2.72 | 10.01 | < 0.001 |
| PLoS Med - New Engl J Med | 10.55 | 4.84 | 23.01 | < 0.001 |

### 6.1.4 Clinical context

The likelihood ratio test revealed a p-value of 0.066 for the
comparison of the full model (journal and time as covariates) and the
reduced model (only time as covariate).

Logistic regression model - clinical context

|  | OR | Lower 95% CI | Upper 95% CI | p-value |
| --- | --- | --- | --- | --- |
| Time | 1.10 | 1.01 | 1.19 | 0.025 |
| JAMA | 1.57 | 0.81 | 3.06 | 0.185 |
| Lancet | 1.80 | 0.91 | 3.55 | 0.093 |
| New Engl J Med | 2.94 | 1.37 | 6.29 | 0.005 |
| PLoS Med | 1.57 | 0.81 | 3.06 | 0.185 |

Logistic regression model with multiple comparisons - clinical
context


|  | OR | Lower 95% CI | Upper 95% CI | Adjusted p-value |
| --- | --- | --- | --- | --- |
| JAMA - BMJ | 1.57 | 0.81 | 3.06 | 0.673 |
| Lancet - BMJ | 1.80 | 0.91 | 3.55 | 0.445 |
| New Engl J Med - BMJ | 2.94 | 1.37 | 6.29 | 0.043 |
| PLoS Med - BMJ | 1.57 | 0.81 | 3.06 | 0.673 |
| Lancet - JAMA | 1.14 | 0.56 | 2.35 | 0.996 |
| New Engl J Med - JAMA | 1.87 | 0.85 | 4.14 | 0.531 |
| PLoS Med - JAMA | 1.00 | 0.49 | 2.02 | 1.000 |
| New Engl J Med - Lancet | 1.64 | 0.73 | 3.67 | 0.753 |
| PLoS Med - Lancet | 0.87 | 0.43 | 1.80 | 0.996 |
| PLoS Med - New Engl J Med | 0.53 | 0.24 | 1.18 | 0.531 |

### 6.1.5 Treatment

The likelihood ratio test revealed a p-value of <0.001 for the
comparison of the full model (journal and time as covariates) and the
reduced model (only time as covariate).

Logistic regression model - treatment

|  | OR | Lower 95% CI | Upper 95% CI | p-value |
| --- | --- | --- | --- | --- |
| Time | 1.08 | 1.02 | 1.16 | 0.015 |
| JAMA | 2.85 | 1.59 | 5.12 | < 0.001 |
| Lancet | 3.81 | 2.11 | 6.88 | < 0.001 |
| New Engl J Med | 3.22 | 1.79 | 5.80 | < 0.001 |
| PLoS Med | 0.86 | 0.47 | 1.60 | 0.641 |

Logistic regression model with multiple comparisons -
treatment


|  | OR | Lower 95% CI | Upper 95% CI | Adjusted p-value |
| --- | --- | --- | --- | --- |
| JAMA - BMJ | 2.85 | 1.59 | 5.12 | 0.004 |
| Lancet - BMJ | 3.81 | 2.11 | 6.88 | < 0.001 |
| New Engl J Med - BMJ | 3.22 | 1.79 | 5.80 | < 0.001 |
| PLoS Med - BMJ | 0.86 | 0.47 | 1.60 | 0.990 |
| Lancet - JAMA | 1.34 | 0.76 | 2.36 | 0.856 |
| New Engl J Med - JAMA | 1.13 | 0.64 | 1.98 | 0.993 |
| PLoS Med - JAMA | 0.30 | 0.17 | 0.55 | < 0.001 |
| New Engl J Med - Lancet | 0.85 | 0.48 | 1.50 | 0.979 |
| PLoS Med - Lancet | 0.23 | 0.12 | 0.41 | < 0.001 |
| PLoS Med - New Engl J Med | 0.27 | 0.15 | 0.49 | < 0.001 |

### 6.1.6 Study name

The likelihood ratio test revealed a p-value of <0.001 for the
comparison of the full model (journal and time as covariates) and the
reduced model (only time as covariate).

Logistic regression model - study acronym

|  | OR | Lower 95% CI | Upper 95% CI | p-value |
| --- | --- | --- | --- | --- |
| Time | 1.13 | 1.03 | 1.24 | 0.008 |
| JAMA | 2.00 | 0.91 | 4.41 | 0.086 |
| Lancet | 6.62 | 3.16 | 13.87 | < 0.001 |
| PLoS Med | 0.90 | 0.37 | 2.21 | 0.821 |

Logistic regression model with multiple comparisons - study
acronym

|  | OR | Lower 95% CI | Upper 95% CI | Adjusted p-value |
| --- | --- | --- | --- | --- |
| JAMA - BMJ | 2.00 | 0.91 | 4.41 | 0.310 |
| Lancet - BMJ | 6.62 | 3.16 | 13.87 | < 0.001 |
| PLoS Med - BMJ | 0.90 | 0.37 | 2.21 | 0.996 |
| Lancet - JAMA | 3.31 | 1.75 | 6.24 | 0.001 |
| PLoS Med - JAMA | 0.45 | 0.20 | 1.01 | 0.214 |
| PLoS Med - Lancet | 0.14 | 0.06 | 0.29 | < 0.001 |

## 6.2 Topic - Results

### 6.2.1 Results mention

Not performed because of low frequencies of articles that mentioned
results in their titles.

### 6.2.2 Quantitative information

Not performed because all articles considered did not have a
quantitative information in the title.

### 6.2.3 Semi-quantitative information

Not performed because of low frequencies of articles with
semi-quantitative information in their titles.

### 6.2.4 Relation

The likelihood ratio test revealed a p-value of <0.001 for the
comparison of the full model (journal and time as covariates) and the
reduced model (only time as covariate).

Logistic regression model - relation

|  | OR | Lower 95% CI | Upper 95% CI | p-value |
| --- | --- | --- | --- | --- |
| Time | 1.01 | 0.94 | 1.07 | 0.858 |
| JAMA | 1.94 | 1.09 | 3.45 | 0.023 |
| Lancet | 0.50 | 0.28 | 0.88 | 0.017 |
| New Engl J Med | 0.28 | 0.15 | 0.52 | < 0.001 |
| PLoS Med | 1.22 | 0.70 | 2.13 | 0.483 |

Logistic regression model with multiple comparisons -
relation


|  | OR | Lower 95% CI | Upper 95% CI | Adjusted p-value |
| --- | --- | --- | --- | --- |
| JAMA - BMJ | 1.94 | 1.09 | 3.45 | 0.154 |
| Lancet - BMJ | 0.50 | 0.28 | 0.88 | 0.120 |
| New Engl J Med - BMJ | 0.28 | 0.15 | 0.52 | < 0.001 |
| PLoS Med - BMJ | 1.22 | 0.70 | 2.13 | 0.956 |
| Lancet - JAMA | 0.26 | 0.14 | 0.46 | < 0.001 |
| New Engl J Med - JAMA | 0.14 | 0.08 | 0.27 | < 0.001 |
| PLoS Med - JAMA | 0.63 | 0.35 | 1.12 | 0.508 |
| New Engl J Med - Lancet | 0.56 | 0.30 | 1.04 | 0.353 |
| PLoS Med - Lancet | 2.43 | 1.38 | 4.31 | 0.019 |
| PLoS Med - New Engl J Med | 4.35 | 2.36 | 8.00 | < 0.001 |

# 7 Logistic regression models - Form

## 7.1 Topic - Methods

### 7.1.1 Abbreviation

The likelihood ratio test revealed a p-value of <0.001 for the
comparison of the full model (journal and time as covariates) and the
reduced model (only time as covariate).

Logistic regression model - abbreviation

|  | OR | Lower 95% CI | Upper 95% CI | p-value |
| --- | --- | --- | --- | --- |
| Time | 1.13 | 1.05 | 1.20 | < 0.001 |
| JAMA | 1.76 | 0.98 | 3.17 | 0.058 |
| Lancet | 2.76 | 1.54 | 4.96 | < 0.001 |
| New Engl J Med | 0.70 | 0.37 | 1.32 | 0.270 |
| PLoS Med | 1.05 | 0.57 | 1.92 | 0.879 |

Logistic regression model with multiple comparisons -
abbreviation


|  | OR | Lower 95% CI | Upper 95% CI | Adjusted p-value |
| --- | --- | --- | --- | --- |
| JAMA - BMJ | 1.76 | 0.98 | 3.17 | 0.320 |
| Lancet - BMJ | 2.76 | 1.54 | 4.96 | 0.006 |
| New Engl J Med - BMJ | 0.70 | 0.37 | 1.32 | 0.804 |
| PLoS Med - BMJ | 1.05 | 0.57 | 1.92 | 1.000 |
| Lancet - JAMA | 1.57 | 0.89 | 2.76 | 0.525 |
| New Engl J Med - JAMA | 0.40 | 0.22 | 0.73 | 0.026 |
| PLoS Med - JAMA | 0.59 | 0.33 | 1.07 | 0.406 |
| New Engl J Med - Lancet | 0.25 | 0.14 | 0.47 | < 0.001 |
| PLoS Med - Lancet | 0.38 | 0.21 | 0.68 | 0.010 |
| PLoS Med - New Engl J Med | 1.49 | 0.80 | 2.80 | 0.719 |

### 7.1.2 Dash

Not performed because of low frequencies of articles that contained a
dash in their titles.

### 7.1.3 Subtitle

The likelihood ratio test revealed a p-value of <0.001 for the
comparison of the full model (journal and time as covariates) and the
reduced model (only time as covariate).

Logistic regression model - subtitle

|  | OR | Lower 95% CI | Upper 95% CI | p-value |
| --- | --- | --- | --- | --- |
| Time | 1.22 | 1.07 | 1.38 | 0.003 |
| JAMA | 0.01 | 0.00 | 0.05 | < 0.001 |
| Lancet | 1.00 | 0.10 | 9.81 | 1.000 |
| New Engl J Med | 0.00 | 0.00 | 0.00 | < 0.001 |
| PLoS Med | 0.59 | 0.08 | 4.59 | 0.616 |

Logistic regression model with multiple comparisons -
subtitle


|  | OR | Lower 95% CI | Upper 95% CI | Adjusted p-value |
| --- | --- | --- | --- | --- |
| JAMA - BMJ | 0.01 | 0.00 | 0.05 | < 0.001 |
| Lancet - BMJ | 1.00 | 0.10 | 9.81 | 1.000 |
| New Engl J Med - BMJ | 0.00 | 0.00 | 0.00 | < 0.001 |
| PLoS Med - BMJ | 0.59 | 0.08 | 4.59 | 0.986 |
| Lancet - JAMA | 111.88 | 20.81 | 601.65 | < 0.001 |
| New Engl J Med - JAMA | 0.03 | 0.01 | 0.12 | < 0.001 |
| PLoS Med - JAMA | 66.27 | 17.28 | 254.11 | < 0.001 |
| New Engl J Med - Lancet | 0.00 | 0.00 | 0.00 | < 0.001 |
| PLoS Med - Lancet | 0.59 | 0.08 | 4.59 | 0.986 |
| PLoS Med - New Engl J Med | 2030.86 | 329.06 | 12533.81 | < 0.001 |

## 7.2 Topic - Conclusion/Discussion

### 7.2.1 Declarative titles

Not performed because all articles considered did not have a
declarative title.

### 7.2.2 Phrasal titles

The likelihood ratio test revealed a p-value of 0.024 for the
comparison of the full model (journal and time as covariates) and the
reduced model (only time as covariate).

Logistic regression model - phrasal title

|  | OR | Lower 95% CI | Upper 95% CI | p-value |
| --- | --- | --- | --- | --- |
| Time | 0.90 | 0.81 | 1.00 | 0.044 |
| JAMA | 0.28 | 0.08 | 0.95 | 0.042 |
| Lancet | 1.10 | 0.47 | 2.60 | 0.828 |
| New Engl J Med | 0.62 | 0.24 | 1.64 | 0.338 |
| PLoS Med | 1.42 | 0.62 | 3.23 | 0.409 |

Logistic regression model with multiple comparisons - phrasal
title


|  | OR | Lower 95% CI | Upper 95% CI | Adjusted p-value |
| --- | --- | --- | --- | --- |
| JAMA - BMJ | 0.28 | 0.08 | 0.95 | 0.243 |
| Lancet - BMJ | 1.10 | 0.47 | 2.60 | 0.999 |
| New Engl J Med - BMJ | 0.62 | 0.24 | 1.64 | 0.870 |
| PLoS Med - BMJ | 1.42 | 0.62 | 3.23 | 0.921 |
| Lancet - JAMA | 3.96 | 1.16 | 13.47 | 0.173 |
| New Engl J Med - JAMA | 2.24 | 0.61 | 8.26 | 0.738 |
| PLoS Med - JAMA | 5.09 | 1.53 | 16.92 | 0.058 |
| New Engl J Med - Lancet | 0.57 | 0.22 | 1.47 | 0.764 |
| PLoS Med - Lancet | 1.29 | 0.57 | 2.89 | 0.973 |
| PLoS Med - New Engl J Med | 2.27 | 0.90 | 5.74 | 0.402 |

### 7.2.3 Question

Not performed because of low frequencies of articles that had a
question mark in their title.

# 8 Comparison with Kerans et al.

We compared our results regarding the items methods mention, patient
population, geography, clinical context, and treatment to those of
Kerans et al. (2016, 2020).

## 8.1 Methods mention

Absolute frequencies - methods mention (Kerans)

|  | Yes | No |
| --- | --- | --- |
| BMJ | 54 | 1 |
| JAMA | 28 | 33 |
| Lancet | 74 | 2 |
| NEJM | 6 | 47 |

Proportions and corresponding 95% CIs - methods mention
(Kerans)

|  | relFreq | Lower 95% CI | Upper 95% CI |
| --- | --- | --- | --- |
| BMJ | 98.2 | 89.0 | 99.9 |
| JAMA | 45.9 | 33.3 | 59.1 |
| Lancet | 97.4 | 90.0 | 99.5 |
| NEJM | 11.3 | 4.7 | 23.7 |

Absolute frequencies - methods mention (our study)

|  | Yes | No |
| --- | --- | --- |
| BMJ | 98 | 2 |
| JAMA | 47 | 53 |
| Lancet | 93 | 7 |
| NEJM | 11 | 89 |

Proportions and corresponding 95% CIs - methods mention (our
study)

|  | relFreq | Lower 95% CI | Upper 95% CI |
| --- | --- | --- | --- |
| BMJ | 98.0 | 92.3 | 99.7 |
| JAMA | 47.0 | 37.0 | 57.2 |
| Lancet | 93.0 | 85.6 | 96.9 |
| NEJM | 11.0 | 5.9 | 19.2 |

P-values from Fisher’s exact tests - methods mention

|  | p-value |
| --- | --- |
| BMJ | 1.000 |
| JAMA | 1.000 |
| Lancet | 0.303 |
| NEJM | 1.000 |

Cramérs V plus corresponding 95% CI and p-values from
Fisher-Freeman-Halton exact tests - methods mention

|  | Cramérs V | Lower 95% CI | Upper 95% CI | p-value |
| --- | --- | --- | --- | --- |
| Kerans et al. | 0.75 | 0.67 | 0.82 | <0.001 |
| Our study | 0.73 | 0.68 | 0.79 | <0.001 |

## 8.2 Patient population

Absolute frequencies - patient population (Kerans)

|  | Yes | No |
| --- | --- | --- |
| BMJ | 21 | 55 |
| JAMA | 18 | 43 |
| Lancet | 15 | 61 |
| NEJM | 16 | 45 |

Proportions and corresponding 95% CIs - patient population
(Kerans)

|  | relFreq | Lower 95% CI | Upper 95% CI |
| --- | --- | --- | --- |
| BMJ | 27.6 | 18.3 | 39.3 |
| JAMA | 29.5 | 18.9 | 42.7 |
| Lancet | 19.7 | 11.8 | 30.8 |
| NEJM | 26.2 | 16.2 | 39.3 |

Absolute frequencies - patient population (our study)

|  | Yes | No |
| --- | --- | --- |
| BMJ | 62 | 38 |
| JAMA | 76 | 24 |
| Lancet | 72 | 28 |
| NEJM | 78 | 22 |

Proportions and corresponding 95% CIs - patient population (our
study)

|  | relFreq | Lower 95% CI | Upper 95% CI |
| --- | --- | --- | --- |
| BMJ | 62.0 | 51.7 | 71.4 |
| JAMA | 76.0 | 66.2 | 83.7 |
| Lancet | 72.0 | 62.0 | 80.3 |
| NEJM | 78.0 | 68.4 | 85.4 |

P-values from Fisher’s exact tests - patient
population

|  | p-value |
| --- | --- |
| BMJ | <.0.001 |
| JAMA | <.0.001 |
| Lancet | <.0.001 |
| NEJM | <.0.001 |

Cramérs V plus corresponding 95% CI and p-values from
Fisher-Freeman-Halton exact tests - patient population

|  | Cramérs V | Lower 95% CI | Upper 95% CI | p-value |
| --- | --- | --- | --- | --- |
| Kerans et al. | 0 | 0 | 0.06 | 0.56 |
| Our study | 0.11 | 0 | 0.22 | 0.065 |

## 8.3 Geography

Absolute frequencies - geography (Kerans)

|  | Yes | No |
| --- | --- | --- |
| BMJ | 12 | 64 |
| JAMA | 9 | 52 |
| Lancet | 16 | 60 |
| NEJM | 9 | 52 |

Proportions and corresponding 95% CIs - geography
(Kerans)

|  | relFreq | Lower 95% CI | Upper 95% CI |
| --- | --- | --- | --- |
| BMJ | 15.8 | 8.8 | 26.4 |
| JAMA | 14.8 | 7.4 | 26.7 |
| Lancet | 21.1 | 12.9 | 32.2 |
| NEJM | 14.8 | 7.4 | 26.7 |

Absolute frequencies - geography (our study)

|  | Yes | No |
| --- | --- | --- |
| BMJ | 31 | 69 |
| JAMA | 16 | 84 |
| Lancet | 17 | 83 |
| NEJM | 9 | 81 |

Proportions and corresponding 95% CIs - geography (our
study)

|  | relFreq | Lower 95% CI | Upper 95% CI |
| --- | --- | --- | --- |
| BMJ | 31.0 | 22.3 | 41.1 |
| JAMA | 16.0 | 9.7 | 25.0 |
| Lancet | 17.0 | 10.5 | 26.1 |
| NEJM | 9.0 | 4.5 | 16.8 |

P-values from Fisher’s exact tests - geography

|  | p-value |
| --- | --- |
| BMJ | 0.022 |
| JAMA | 1.000 |
| Lancet | 0.560 |
| NEJM | 0.446 |

Cramérs V plus corresponding 95% CI and p-values from
Fisher-Freeman-Halton exact tests - geography

|  | Cramérs V | Lower 95% CI | Upper 95% CI | p-value |
| --- | --- | --- | --- | --- |
| Kerans et al. | 0 | 0 | 0.06 | 0.724 |
| Our study | 0.18 | 0.04 | 0.28 | 0.002 |

## 8.4 Clinical context

Absolute frequencies - clinical context (Kerans)

|  | Yes | No |
| --- | --- | --- |
| BMJ | 61 | 15 |
| JAMA | 50 | 11 |
| Lancet | 63 | 13 |
| NEJM | 55 | 6 |

Proportions and corresponding 95% CIs - clinical context
(Kerans)

|  | relFreq | Lower 95% CI | Upper 95% CI |
| --- | --- | --- | --- |
| BMJ | 80.3 | 69.2 | 88.2 |
| JAMA | 82.0 | 69.6 | 90.2 |
| Lancet | 82.9 | 72.2 | 90.2 |
| NEJM | 90.2 | 79.1 | 95.9 |

Absolute frequencies - clinical context (our study)

|  | Yes | No |
| --- | --- | --- |
| BMJ | 73 | 27 |
| JAMA | 81 | 19 |
| Lancet | 83 | 17 |
| NEJM | 89 | 11 |

Proportions and corresponding 95% CIs - clinical context (our
study)

|  | relFreq | Lower 95% CI | Upper 95% CI |
| --- | --- | --- | --- |
| BMJ | 73.0 | 63.0 | 81.2 |
| JAMA | 81.0 | 71.7 | 87.9 |
| Lancet | 83.0 | 73.9 | 89.5 |
| NEJM | 89.0 | 80.8 | 94.1 |

P-values from Fisher’s exact tests - clinical context

|  | p-value |
| --- | --- |
| BMJ | 0.289 |
| JAMA | 1.000 |
| Lancet | 1.000 |
| NEJM | 1.000 |

Cramérs V plus corresponding 95% CI and p-values from
Fisher-Freeman-Halton exact tests - clinical context

|  | Cramérs V | Lower 95% CI | Upper 95% CI | p-value |
| --- | --- | --- | --- | --- |
| Kerans et al. | 0 | 0 | 0.04 | 0.431 |
| Our study | 0.12 | 0 | 0.22 | 0.036 |

## 8.5 Treatment

Absolute frequencies - treatment (Kerans)

|  | Yes | No |
| --- | --- | --- |
| BMJ | 28 | 48 |
| JAMA | 27 | 45 |
| Lancet | 55 | 21 |
| NEJM | 35 | 26 |

Proportions and corresponding 95% CIs - treatment
(Kerans)

|  | relFreq | Lower 95% CI | Upper 95% CI |
| --- | --- | --- | --- |
| BMJ | 36.8 | 26.3 | 48.7 |
| JAMA | 44.3 | 31.8 | 57.5 |
| Lancet | 72.4 | 60.7 | 81.7 |
| NEJM | 57.4 | 44.1 | 69.7 |

Absolute frequencies - treatment (our study)

|  | Yes | No |
| --- | --- | --- |
| BMJ | 30 | 70 |
| JAMA | 55 | 45 |
| Lancet | 62 | 38 |
| NEJM | 58 | 42 |

Proportions and corresponding 95% CIs - treatment (our
study)

|  | relFreq | Lower 95% CI | Upper 95% CI |
| --- | --- | --- | --- |
| BMJ | 30.0 | 21.5 | 40.1 |
| JAMA | 55.0 | 44.8 | 64.9 |
| Lancet | 62.0 | 51.7 | 71.4 |
| NEJM | 58.0 | 47.7 | 67.7 |

P-values from Fisher’s exact tests - treatment

|  | p-value |
| --- | --- |
| BMJ | 0.418 |
| JAMA | 0.198 |
| Lancet | 0.197 |
| NEJM | 1.000 |

Cramérs V plus corresponding 95% CI and p-values from
Fisher-Freeman-Halton exact tests - treatment

|  | Cramérs V | Lower 95% CI | Upper 95% CI | p-value |
| --- | --- | --- | --- | --- |
| Kerans et al. | 0.29 | 0.17 | 0.38 | <0.001 |
| Our study | 0.24 | 0.13 | 0.33 | <0.001 |
