# Supplementary material for: Content and form of original research articles in general major medical journals
Source: PLoS One. 2023 Jun 28;18(6):e0287677. doi: 10.1371/journal.pone.0287677 (PMC10306213; doi:10.1371/journal.pone.0287677)
Supplement: Attachment — Submitted filename: Response_to_Reviewers_V01.pdf [file pone.0287677.s004.pdf]

Point-by-point reply to the reviewer comments

PLOS ONE PONE-D-23-07021
"Title Content and Form of Original Research Articles in General Major Medical Journals"

We are very grateful to all reviewers for their feedback and helpful comments which have led to considerable improvement of our manuscript. Reviewer comments are highlighted in blue, and answers to their comments are provided in black in this document. Added and/or altered text is given in italic. These changes are highlighted in the revised manuscript. In addition, we provide a clean version of our manuscript.

Journal requirements

When submitting your revision, we need you to address these additional requirements.
Please ensure that your manuscript meets PLOS ONE's style requirements, including those for file naming. The PLOS ONE style templates can be found at https://journals.plos.org/plosone/s/file?id=wjVg/PLOSOne_formatting_sample_main_body.pdf and https://journals.plos.org/plosone/s/file?id=ba62/PLOSOne_formatting_sample_title_authors_affiliations.pdf
We revised our manuscript in accordance with the PLOS ONE style requirements including those for file naming.

Thank you for stating the following in the Competing Interests section: "There are no patents, products in development or marketed products to declare. AZ is a licensed Tim Albert trainer and has held several courses in the past based on Albert's concept."
Please confirm that this does not alter your adherence to all PLOS ONE policies on sharing data and materials, by including the following statement: "This does not alter our adherence to PLOS ONE policies on sharing data and materials." (as detailed online in our guide for authors http://journals.plos.org/plosone/s/competing-interests). If there are restrictions on sharing of data and/or materials, please state these. Please note that we cannot proceed with consideration of your article until this information has been declared.
Please include your updated Competing Interests statement in your cover letter; we will change the online submission form on your behalf.
We updated the Competing Interests statement in the cover letter.

In your Data Availability statement, you have not specified where the minimal data set underlying the results described in your manuscript can be found. PLOS defines a study's minimal data set as the underlying data used to reach the conclusions drawn in the manuscript and any additional data required to replicate the reported study findings in their entirety. All PLOS journals require that the minimal data set be made fully available.

For more information about our data policy, please see http://journals.plos.org/plosone/s/data-availability.

"Upon re-submitting your revised manuscript, please upload your study's minimal underlying data set as either Supporting Information files or to a stable, public repository and include the relevant URLs, DOIs, or accession numbers within your revised cover letter. For a list of acceptable repositories, please see http://journals.plos.org/plosone/s/data-availability#loc-recommended-repositories.

Any potentially identifying patient information must be fully anonymized.

Important: If there are ethical or legal restrictions to sharing your data publicly, please explain these restrictions in detail. Please see our guidelines for more information on what we consider unacceptable restrictions to publicly sharing data: http://journals.plos.org/plosone/s/data-availability#loc-unacceptable-data-access-restrictions. Note that it is not acceptable for the authors to be the sole named individuals responsible for ensuring data access.

We will update your Data Availability statement to reflect the information you provide in your cover letter.

We included the Data Availability statement in the cover letter.

Please include captions for your Supporting Information files at the end of your manuscript, and update any in-text citations to match accordingly. Please see our Supporting Information guidelines for more information: http://journals.plos.org/plosone/s/supporting-information.

We included captions for our Supporting Information Files at the end of our manuscript and updated all in-text citations.

Comment Editor

The major concerns from the reviewers include the sample size, sample selection, and writing style.

We are grateful for your summary about the major concerns raised by the reviewers. We have revised our manuscript along your summary comments and all reviewer comments, and we would be very happy if you and all reviewers now find our work to be acceptable for publication in PLOS ONE.

Reviewer 1

This article analyses the titles of articles published in a series of medical journals over time. It is interesting for a consideration of how naming practices affect discoverability and use of research material.

We thank the reviewer for the important points raised below. These points are now addressed in our manuscript.

There are, though, a few aspects that need revision:
First, the title of this paper makes reference to “high ranked” medical journals, but there is no definition anywhere of how this ranking is constructed or to what ranking you are referring.
We fully agree with the reviewer that we did not provide a definition of “high ranked” medical journals. We also switched between “high ranked medical journals” and “general (major) medical journals”. We have changed all concerned text passages to “general major medical journals”, including the title.

Second, the sample size of 500 is relatively small and limits the general applicability of the findings, as the paper notes. I am unclear as to whether this is too small to be useful/generalizable.
The main aim of our work was to investigate trends over time. In general, regression models have a sufficient sample for a single independent variable, such as time, if $n \approx 50$. Specifically, for a weak effect size of $R^2 = 0.14$, the required sample size is 51.
In case of a weak effect size of Cohen’s f with $f^2 = R^2 / (1 – R^2) = 0.14$, the required sample size is 403 to achieve a power of 80%. A sample size of 500 as used in our work yields a power of 87.75% at a significance level of 5%. We have added a section on sample size considerations in the manuscript.
The corresponding R code is provided below. We would be happy to add the code to the manuscript.

```
library(pwr)
results = pwr.f2.test(u=1, power=0.8, f2=0.14/(1-0.14), sig.level=0.05)
n = ceiling(results$v+1+1)
n

results = pwr.f2.test(u=1, power=0.8, f2=0.14^2, sig.level=0.05)
n = ceiling(results$v+1+1)
n

results = pwr.f2.test(u=1, power=0.8775, f2=0.14^2, sig.level=0.05)
n = ceiling(results$v+1+1)
n
```

However, we have to admit that the sample size was too low for other studies, such as the citation frequency in relation to some of the title characteristics. We calculated that more than 1000 article titles would need to be studied.
We have substantially revised the corresponding part in the Discussion. It now reads:

> *A further limitation is the sample size of 500 articles, i.e., 10 articles per journal and year. With a sample size substantially larger than 1000 articles we would have been*

able to study the association of title characteristics with citation counts. For example, the total sample size of our previous study, which was based on an automated search was 8096 articles [1]. With 500 articles, 95% confidence intervals are approximately 4 times larger ($\sqrt{8096} / \sqrt{500} = 4.02$), and many results, such as the association between the number of citations would not have been significant. The sample size used in this study is approximately twice that of [2, 3], and this study with 500 articles was powered to reliably detect trends over time.

Third, some of the limitations could have been overcome with different computational methods. For example, on page 14 you state that the maps package that you used was not able correctly to identify many locations in article headings. However, other named-entity recognition tools would certainly do a better job of this. For instance, Amazon Comprehend or SageMaker could be appropriate tools here.

The commercial tools suggested by the reviewer would be interesting not only for the study of geographic information but also for the analyses of other article characteristics, such as the number of words per paragraph or the number of paragraphs. In our work, we only focused on "does the title contain any geographic information", a minimalistic query. By analyzing this question by hand, we assumed to be more precise than any available automatic tool could be. In our previous work [1], we used the freely available `maps` package, and given our results it is debatable whether the choice of this package was the best one. Because of your comment and a comment by reviewer 2, we have restructured the manuscript on this topic, and the text elements added to the Methods read:

Recently, we performed an analysis after an automatic search for country and city mentioning in the title by the use of the R package `maps` [1], and we did not expect substantial differences to our hand search.

The subsection in the Results reads:

The comparison of our hand search on the mentioning of geographic information revealed substantial differences to the automated search with the R package `maps` [1].

In detail, respectively, 31% vs. 13% for BMJ, 16% vs. 3% for JAMA, 17% vs. 9% for the Lancet, 9% vs. 3% for the NEJM and 52% vs. 29% for PLOS articles contained any geographical information in their titles for the hand and automatic search. The automated search thus led to fewer titles with any geographic information.

Finally, the paragraph in the Discussion reads:

We did not expect different journal-specific frequencies regarding the geographic information in the title compared to our recent work [1], in which we performed an automatic search for country and city mentioning in the title by the use of the R package `maps` *[1]. However, frequencies differed substantially. The automated search led to fewer titles with any geographic information. For example, the* `maps` *package did not contain countries, such as 'England', continents, abbreviation, such as 'U.S.', or terms, such as 'English'. The main reasons for the discrepancies were for the use of country-specific abbreviations and additional country-specific terms. However, other tools or packages might have been more appropriate for the geographical query than the* `maps` *package.*

Fourth, the language needs careful checking throughout. For instance: "can only adequately [be] measured"; "articles meaning no sentence and no question"; "we did neither observe" → "we observed neither"; "almost the half" → "almost half".
We thank the reviewer for this important remark and the provided examples. We checked the language throughout the manuscript.

Fifth, and perhaps most significantly, it would be helpful for the conclusions of this paper to interpret the findings more closely. Why have these changes that you find occurred?
We thank the reviewer for this comment. Accordingly, we have revised the paragraph on declamatory titles in the Discussion, which now reads:

Some content criteria were mainly not or rarely used in all considered journals, such as a dash, mentioning of results, using a declarative title, or a question mark. This was in contrast to Paiva, Lima (4) who showed for PLOS and BMC journals that approximately 40% of the articles mentioned the results, and such articles were more frequently cited than work mentioning methods. In our study, only 6 articles out of 500 mentioned results in the title, while 344 out of the 500 articles mentioned of methods. Our findings are in line with general guidelines that declamatory titles, i.e., titles that give study results should be avoided [5]; see, e.g., instructions to authors for the Lancet. Authors should thus avoid providing quantitative or semi-quantitative information in the title. In fact, since the title is a one-line summary, the conclusions could be spread out into the world without reading at least the abstract or the full text of the article. Aleixandre-Benavent and colleagues go a step further and provide recommendations what a title should contain, and how it should not be constructed [6].

Furthermore, we discuss the use of subtitles, which have changed over time. The corresponding text reads:

Subtitles are now more frequently used than a decade ago. Furthermore, the mentioning of methods increased in the 10 years from 2011 to 2020. This change in the title may be caused by the increased use of reporting guideline, such as the CONSORT statement [7], which states that a randomized controlled trial should be identifiable as randomized in the title. The instructions for authors of all considered journals state that subtitles should be used for reporting the study design and/or authors should follow the respective reporting guidelines of their study. In fact, authors should look out a copy of the target journal and identify its preferences [8].

What does it mean that subtitles are now more common?

Many original articles in the general major medical journals are concerned with randomized controlled trials (RCTs) and, according to the CONSORT 2010 statement, a RCT should be identifiable as such from its title (Schulz et al., 2010). In practice to overcome this, titles mainly consist in at least two parts with a subtitle when a RCT was conducted. This is most done by using the colon as the separator in a title to split the title into multiple parts (Heßler & Ziegler, 2022, Kerans et al., 2016, Kerans et al., 2020). However, the main impact of the CONSORT guideline was the endorsement by journals, which has improved clinical trial reporting (Hopewell et al., 2022). In fact, the investigated journals state in their instructions for authors how to construct a title for the report of a clinical study. While the NEJM only refers to the CONSORT statement, the other four journals are more precise and clearly state "use subtitles for describing the study design". We added these two points in our discussion section (see your previous comment):

Subtitles are now more frequently used than a decade ago. Furthermore, the mentioning of methods increased in the 10 years from 2011 to 2020. This change in the title may be caused by the increased use of reporting guidelines, such as the CONSORT statement [7], which states that a randomized controlled trial should be identifiable as randomized in the title. The instructions for authors of all considered journals state that subtitles should be used for reporting the study design and/or authors should follow the respective reporting guidelines of their study. In fact, authors should look out a copy of the target journal and identify its preferences [8].

How does discoverability work in each of the title types to which you refer?

We thank the reviewer for this important comment. To make the evaluation of the title items (form and content) more comprehensible for the reader, we have included two examples of article titles. For these two title examples, we added the evaluation of all items into Table 1; please see Table 1 in the revised manuscript. Additionally, we provided the following sentence in the Methods:

One reviewer asked for the discoverability in each of the title items, therefore, we provided two examples of article titles with the result of our evaluation in Table 1.

Finally, you open by stating that the prime driver of picking a good title is so that you can pick up citations and have career progression. This seems a very cynical way of thinking about how to title articles. Scientists and medics should use titles that accurately reflect the content of the work and allow others easily to find and re-use their research. I would suggest amending this opening to incorporate such a stance.

Of course, researchers should use titles that accurately reflect the content of their work and allow others easily to find and re-use their research. In fact, when scientists search databases for references, e.g. Medline, the title plays a major role. In Medline, article titles are not categorized directly. Instead, they are indexed as part of the entire article and categorized based on the topics and content covered in the article, primarily done by using MeSH terms. These terms are selected through manual assignment by indexers who carefully read and analyze the article to identify its most important topics and content. Although the title of an article has no direct categorization, it serves to determine the relevance of the article to specific MeSH terms. However, the accurate use of MeSH terms can help an article be found more easily by other researchers when they search for articles on a particular topic. If an article uses relevant and comprehensive MeSH terns, it is more likely to be cited by other researchers. We added the suggestion of the reviewer in the second paragraph of our introduction. The relevant text reads:

Especially the title has been proposed as a modifiable component of a research article [1, 9, 10]. Researchers should use titles that accurately reflect the content of their work and allow others easily to find and re-use their research [11]. Most research has focused on the form of article titles because these analyses could be performed automatically and are not very time-consuming [1, 12, 13].

Reviewer 2

The paper titled "Title Content and Form of Original Research Articles in High-Ranked Medical Journals" investigates the differences of title content and form between papers in the medical field and their changes over time. Overall, the paper is well-written and well-argued. The methodology is adequate, and there are an overall coherence and relation to the scope of publication in the Plos One journal. In addition, this manuscript addresses a very interesting issue about the analysis of titles and does so in a very competent technical way. It Is worth mentioning that data and R code are shared.
We thank the reviewer for this very kind feedback. We are glad to have the opportunity to provide more information on the points raised by the reviewer.

I do however have a major issue (which in fact, is a minor one). The authors did a huge effort in sharing all the data; however, the results section sometimes it's difficult to follow (the reader should go back and forth checking the tables). I think some (introductory) sentences in some parts of the manuscript will benefit the readability of the text (see my suggestions below). I will go slightly more into detail with them in the position-specific comments below.
We fully agree to this comment. A huge amount of results was provided to answer our prespecified research questions. As suggested by the reviewer, we used some introductory sentences in some parts of the manuscript to increase the readability of the article.

Keywords:
I think the paper would benefit by including some keywords related to the titles (e.g. research article titles or titles). I have reservations about the use of 'impact' (I think the authors are not analysing the impact of the papers, e.g. citation impact).
We followed the recommendation of the reviewer, and we updated our keywords, i.e., we removed "impact" and added "article title" to our keyword list.

Introduction
The introduction and background is ok, providing the necessary information leading to the purpose of the study. However, I think the authors could expand a bit more (there are more studies on the topic). See my suggestions below.
We added the suggestions of the reviewer listed below into our introduction and revised the introduction. Please see the corresponding comments below.

P10|Line 56. Indicate that the EQUATOR Network is referred to the reporting health research (e.g. Enhancing the QUAlity and Transparency Of health Research (EQUATOR) Network).
Done.

P10|Line 33 (and P11|Line 83). "content has rarely been investigated beyond title length". I slightly disagree with this statement. From a bibliometric perspective, there are many articles that analyse impact (e.g. effect on citations, downloads), sentence types (e.g. informative), the information the author wants to include, and in which order, among others. I would like to suggest the following papers (not included by the authors):

• Aleixandre-Benavent, R., Montalt-Resurecció, V., & Valderrama-Zurián, J. C. (2014). A descriptive study of inaccuracy in article titles on bibliometrics published in biomedical journals. Scientometrics, 101(1), 781–791. https://doi.org/10.1007/s11192-014-1296-5.
• Ball, R. (2009). Scholarly communication in transition: The use of question marks in the titles of scientific articles in medicine, life sciences and physics 1966–2005. Scientometrics, 79(3), 667–679. https://doi.org/10.1007/s11192-007-1984-5.
• Busch-Lauer, I.-A. (2000). Titles of English and German research papers in medicine and linguistics theses and research articles. In A. Trosborg (Ed.), Analysing professional genres (pp. 77–94). John Benjamins Publishing Company. https://doi.org/10.1075/pbns.74.08bus
• Buter, R. K., & van Raan, A. F. J. (2011). Non-alphanumeric characters in titles of scientific publications: An analysis of their occurrence and correlation with citation impact. Journal of Informetrics, 5(4), 608–617. https://doi.org/10.1016/j.joi.2011.05.008.
• Haggan, M. (2004). Research paper titles in literature, linguistics and science: Dimensions of attraction. Journal of Pragmatics, 36(2), 293–317. https://doi.org/10.1016/S0378-2166(03)00090-0
• Nagano, R. L. (2015). Research article titles and disciplinary conventions: A corpus study of eight disciplines. Journal of Academic Writing, 5(1), 133–144. https://doi.org/10.18552/joaw.v5i1.168
• Pearson, W. S. (2021). Quoted speech in linguistics research article titles: patterns of use and effects on citations. Scientometrics, 126(4), 3421-3442.

We thank the reviewer for the provided references, and we have added more than half of them.
Aleixandre-Benavent et al. → Discussion, Ball → Introduction, Busch-Lauer et al. → not included, Buter → Introduction + Discussion, Haggan → Introduction + Discussion, Nagano → not added, Pearson → not added

P10|Line 75. Worth mentioning the Web of Science (1997) which includes the title field tag.

We included the Web of Science. The sentence now reads:

> *For example, PubMed was first released in 1996, the Web of Science is online since 1997 and Google Scholar started not earlier than in 2004.*

P11|Line 79. Indicate the acronym (Standards for Reporting of Diagnostic Accuracy (STARD)). When the authors mention 'at least two observers should do an independent evaluation where applicable". Are referring to the article title? (not clear)

First, we inserted the long term for the abbreviation of the STARD statement. Second, the point regarding the need of at least two observers referred to the statement, that an evaluation through two raters can also be time-consuming. We revised the text passage, which now reads:

> *Another reason could be that these evaluations have to be made manually, and they are thus time-consuming [14]. An additional time-consuming factor could be that guidelines such as the Standards for Reporting of Diagnostic Accuracy (STARD) statement [15] strongly recommend that at least two observers should do an independent evaluation where applicable.*

P11|Line 84. Correct typo "(2020)compared".

Corrected.

Methods

P12|106-108. The authors mention "Articles being listed with an abstract remained in the data set, while articles only listed in the Web of Science were excluded.". I suggest indicating the number of papers. Was the abstract used for any purpose?

The data generating steps, i.e., search, download and identification of original research articles in PubMed and Web of Science were described in detail in our other work (Heßler & Ziegler, 2022). There, we wrote:

"For example, in one step we tried to identify original articles by structured abstracts with the PubMed search term "bmj" [Journal] AND "hasstructuredabstract" [All Fields] AND 2017 dp]. While the number of articles with a structured abstract was between 150 and 160 for the years 2011 and 2020, it was just 29 for the year 2017, without a single article having a structured abstract between March and November. However, with the search term "bmj" [Journal] AND "hasabstract" [All Fields] AND 2017 [dp] we were able to identify articles with a structured abstract. When we looked at these articles in detail, we identified the abstract structure and noted that some formatting signs, such as end of

line, feed forward seemed to be missing. In our opinion, these relevant characters were not transmitted to the National Library of Medicine so that the abstracts were not categorized as "hasstructuredabstract". We made the BMJ aware of this formatting issue (personal communication)."

This issue only came up due to the fact that we downloaded the abstract of articles in our Web of Science search. The abstract was only used for the identification of original articles, not for other purposes. Raised by the reviewer comment, we provided some numbers regarding the number of papers. In detail, 8396 articles were identified in Medline, and 10267 articles in the Web of Science. After cleaning the data, 8096 articles were available. In our opinion, this are the most important numbers. The text in the methods section now reads:

> *… we reduced available information to journal name, article title, PMID, abstract for the identification of original research articles, DOI and publication date. Both PMID and DOI were used to merge articles identified in Medline (n=8396) and the Web of Science (n=10267). Articles being listed with an abstract remained in the data set, while articles only listed in the Web of Science were excluded. Articles being only downloaded in the Medline files were checked whether they were indeed original research articles. If not, they were excluded as well. After data cleaning, a set of 8096 articles was available.*

P12|111. Indicate in this section that ten original articles per year (100 articles per journal) were randomly chosen.

We revised the text in the methods section to make it clearer to the reader. The text now reads:

> *To investigate title content and form, we randomly selected 500 original articles from the years 2011 to 2020. The random selection was done with stratification by journal and year so that ten original articles per year (100 articles per journal) were randomly chosen.*

Results

There is a lot of information in this section (Tables and supplementary material), which allows the reproducibility of the findings. However, I think some introductory sentences will benefit the readability of the text (see my suggestions below).

We revised our manuscript regarding the suggestions of the reviewer mentioned below into our results and revised the results section. Please see the corresponding comments below.

P14|159-160. Although the information is in the Supplementary Material, I suggest introducing a few words (just one or two sentences) about Table 2 (or Descriptive Statistics).

We revised the first paragraph of the results as follows:

> *A total of 500 randomly selected original research articles from 5 medical journals were analyzed regarding the selected title items (see* ***Table 1)****. In* ***Table 2****, the descriptive statistics, i.e., absolute and relative frequencies for all title properties over the years are shown, respectively for each journal.*

In Table 3, the Plos Medicine journal information is missing in the table.

Thank you for this important hint. Indeed, the inclusion of PLOS Medicine information is not possible. Kerans et al. (2016, 2020) did not investigate PLOS Medicine, so that our results could not be compared to their results. However, raised by the reviewer comment, we updated the description of Table 3 to make clear that PLOS Medicine could not be analyzed due to that fact. We added to the description the following hint:

> *Results of PLOS Medicine titles are missing because Kerans et al. did not examine article titles of this journal.*

P14|159-160 "About half of the PLOS titles (52%) contained any geographic information": missing this information in Table 3 (or indicate the Supplementary table in which this information is displayed).

Related to the comment above, this information can not be added in Table 3. Thus, we added the hint that PLOS Medicine could not be included in our comparison analyses. However, we referred to Table 2 where the descriptive results for all considered items are shown. Furthermore, motivated by the reviewer comment, we added this topic to both the Methods and the Results section.

The paragraph added in the Methods reads:

> *Recently, we performed an analysis after an automatic search for country and city mentioning in the title by the use of the R package* `maps` *[1], and we did not expect substantial differences to our hand search.*

The subsection in the Results reads:

> *The comparison of our hand search on the mentioning of geographic information revealed substantial differences to the automated search with the R package maps [1].*

In detail, respectively, 31% vs. 13% for BMJ, 16% vs. 3% for JAMA, 17% vs. 9% for the Lancet, 9% vs. 3% for the NEJM and 52% vs. 29% for PLOS articles contained any geographical information in their titles for the hand and automatic search. The automated search thus led to fewer titles with any geographic information.

Finally, the paragraph in the Discussion reads:

We did not expect different journal-specific frequencies regarding the geographic information in the title compared to our recent work [1], in which we performed an automatic search for country and city mentioning in the title by the use of the R package `maps` *[1]. However, frequencies differed substantially. The automated search led to fewer titles with any geographic information. For example, the maps package did not contain countries, such as 'England', continents, abbreviation, such as 'U.S.', or terms, such as 'English'. The main reasons for the discrepancies were for the use of country-specific abbreviations and additional country-specific terms. However, other tools or packages might have been more appropriate for the geographical query than the maps package.*

P16|203. Correct typo 'ofKerans'.
We checked that. The space was there, but it seems to be missing because of the font type.

P16|207. Here, the authors mention the Results/Relation (and not the previous ones, i.e. Results mention, Quantitative information or Semi-quantitative information). I suggest an introductory sentence indicating that 'In terms of results, etc'. And also pointing out that the other previous items were rarely used.
As raised by the reviewer, we included introductory sentences for each "topic" (see Table 1) of title content or title form.

P16|214. Regarding this information (24%), indicate in brackets Table 2 (or 3.2. Supplementary Table)
We added the reference to Table 2.

P16|224. Indicate that refers to the discussion/conclusion part.
Done. We changed the word "substantial" to "significant".

Discussion/Conclusions
Line 19|278-279. Another aspect that should be considered is the title length allowed by each journal (number maximum of words). Also there is of interest the recommendations from the journals (e.g. in the author guidelines). In some journals there is some criteria

such as ‘Specific, descriptive, concise, and comprehensible to readers outside the field’ (Plos One), whereas in others it is suggested to include a subtitle (e.g. https://jamanetwork.com/journals/jamanetworkopen/pages/instructions-for-authors).
Title length is an important factor of the title which we investigated in detail in our former work (Heßler & Ziegler, 2022). So, this was not focus of our current work. However, we carefully checked the instructions for authors of all journals. Indeed, information regarding the title differs between the journals. For BMJ, the title should be informative and, for research papers, a subtitle with the study design should be used. NEJM only refers to the CONSORT statement. Lancet states on his website that non-declamatory titles including the trials descriptor should be provided. The other two journals provide more information on the title. In JAMA, a title should be concise, specific, informative, and not longer than 150 characters. For scientific manuscripts, general titles, declarative titles, titles that include the direction of study results, or questions as titles should not be used. For report of clinical trials, the type of the study should be included in a subtitle. PLOS states "specific, descriptive, concise, and comprehensible to readers outside the field", not longer than 250 characters and use subtitles for clinical studies. As suggested by the reviewer, we added the reference to the instructions for authors. The text now reads:

> *Our work focused on the general major medical journals plus the online only journal PLOS. Between the printed journals, there were substantial differences regarding the content of article titles [1]. One of the reasons could be in the instructions for authors, which differ in the provided information on the construction of a title.*

Line 20 |321. Another limitation is the variables considered (e.g. some other studies analyse other non-alphanumeric elements such as exclamations, other criteria for the content, etc.)
We thank the reviewer for this point. We added the limitation regarding the variables considered into our discussion. The text reads:

> *Another limitation of our work is that additional variables could have been considered, e.g., more complex title content [6, 11, 16].*

Line21|333. A sentence about further research could be included.
We now provided some aspects for future research. The paragraph reads:

For further research, it could be interesting to investigate more specific journals to compare them to the general medical journals. Furthermore, it would be of interest to analyze, the effect of title content properties on citation frequencies. Therefore, more article titles reduced to a subset of the items considered here will be needed.

Reviewer 3

We thank the reviewer for raising the following comments.

There was a desire to look at characteristics within journals over time but use of only 10 articles per year seems subject to selection bias for this research question. What was the power consideration here?

The main aim of our work was to investigate trends over time. In general, regression models have a sufficient sample for a single independent variable, such as time, if n ≈ 50. Specifically, for a weak effect size of $R^2 = 0.14$, the required sample size is 51.

In case of a weak effect size of Cohen's f with $f^2 = R^2 / (1 – R^2) = 0.14$, the required sample size is 403 to achieve a power of 80%. A sample size of 500 as used in our work yields a power of 87.75% at a significance level of 5%. We have added a section on sample size considerations in the manuscript.

The corresponding R code is provided below. We would be happy to add the code to the manuscript.

```
library(pwr)
results = pwr.f2.test(u=1, power=0.8, f2=0.14/(1-0.14), sig.level=0.05)
n = ceiling(results$v+1+1)
n

results = pwr.f2.test(u=1, power=0.8, f2=0.14^2, sig.level=0.05)
n = ceiling(results$v+1+1)
n

results = pwr.f2.test(u=1, power=0.8775, f2=0.14^2, sig.level=0.05)
n = ceiling(results$v+1+1)
n
```

The power of our study was in our opinion thus not limited by the number of articles per year and journal.

It is not clear how the titles were evaluated. Was it an automated program or each one manually? The samples size is small enough that manual adjudication is possible.

Indeed, we missed to state that we evaluated all items by hand in the methods section of the manuscript. We added the following sentence in the first paragraph of the "Evaluation of title content and form" section:

> *... All article titles were evaluated manually by two raters. ...*

Line 115. This is confusing. Results are not given for harmonization of classification of various title attributes.

Unfortunately, we are not able to understand this comment. We would like to address this point but need further instructions.

Reporting of ORs is confusing. For example on line 170, what is the OR for? The proportion listing method per year? Similar in line 188 – you say the rate varied over time, but you used logistic regression assuming an increase over time?

We add the interpretation of the ORs in the "Statistics" section of the methods. The part about the logistic regression now reads:

> *The effect of time regarding the specific title properties was investigated by logistic regression models, if appropriate. Post hoc comparisons for the identification of homogeneous subgroups were performed using Tukey's HSD. Associations between title properties and the journals were analyzed using likelihood ratio tests. Effect estimates, i.e., odds ratios and corresponding 95% CI were reported for all analyses, and the journal BMJ was used as reference category. An odds ratio of x.x being greater than 1 indicates an x.x fold increased chance containing the specific item for an one-year difference adjusted for the variable journal.*

The results section includes discussion points (like line 202).

We are grateful to the reviewer for this comment. We have added the statement that one aim of our work was the comparison with Kerans et al. (2016, 2020).

> *We also compared our findings to those of Kerans et al. [2, 3].*

We thus consider comparisons with Kerans et al. that are made in the Results section to be results statements rather than discussion statements.

Were any findings linked back to author instructions for each journal? These often dictate title content.

This is a good point which has to be included in the manuscript. A similar comment was raised by reviewer number 2. Some information on how to construct the title in the instructions for authors is provided, for details, please, see our response to reviewer 2. However, we missed to link our results with the recommendations of the journals. We have now added this part in the discussion. The text reads:

> *Our results are in line with the recommendations from the journal-specific instructions for authors, except NEJM. The NEJM does not follow the CONSORT statement using subtitles for randomized controlled trials, see also [1]. For the other four journals, the mentioning of the study design or the type of analysis is almost always done using subtitles as recommended. Furthermore, our results for JAMA using no declarative titles, no results mentioning or using questions in the title match with its recommendations.*

Linking the metrics assessed to citation counts would add an important dimension to the significance of this research.

This is absolutely true. However, more article titles would be necessary to investigate the effect of title content properties on the citation frequencies. We have described this point in our limitation and also include a paragraph about future research to do these analyses. The text reads:

> *A further limitation is the sample size of 500 articles, i.e., 10 articles per journal and year. With a sample size substantially larger than 1000 articles we would have been able to study the association of title characteristics with citation counts. For example, the total sample size of our previous study, which was based on an automated search was 8096 articles [1]. With 500 articles, 95% confidence intervals are approximately 4 times larger (√8096 / √500 = 4.02), and many results, such as the association between the number of citations would not have been significant. The sample size used in this study is approximately twice that of [2, 3], and this study with 500 articles was powered to reliably detect trends over time.*

There is too much repetition of p values in the Discussion. I assume these were not presenting new analyses not shown in the results. It is not appropriate.

p-values deleted from the Discussion.

Line 312 – they do report new analyses. It should be part of the study of not (unless published elsewhere).

Because of the reviewer comment we have added a paragraph on this comparison between the manual and the automated search in the Methods.

The paragraph added in the Methods reads:

> *Recently, we performed an analysis after an automatic search for country and city mentioning in the title by the use of the R package* `maps` *[1], and we did not expect substantial differences to our hand search.*

The subsection in the Results reads:

> *The comparison of our hand search on the mentioning of geographic information revealed substantial differences to the automated search with the R package* `maps` *[1].*
>
> *In detail, respectively, 31% vs. 13% for BMJ, 16% vs. 3% for JAMA, 17% vs. 9% for the Lancet, 9% vs. 3% for the NEJM and 52% vs. 29% for PLOS articles contained any*

geographical information in their titles for the hand and automatic search. The automated search thus led to fewer titles with any geographic information.

Finally, the paragraph in the Discussion now reads:

We did not expect different journal-specific frequencies regarding the geographic information in the title compared to our recent work [1], in which we performed an automatic search for country and city mentioning in the title by the use of the R package `maps` *[1]. However, frequencies differed substantially. The automated search led to fewer titles with any geographic information. For example, the* `maps` *package did not contain countries, such as 'England', continents, abbreviation, such as 'U.S.', or terms, such as 'English'. The main reasons for the discrepancies were for the use of country-specific abbreviations and additional country-specific terms. However, other tools or packages might have been more appropriate for the geographical query than the* `maps` *package.*

The Discussion is too long.

Previously, we showed that PLOS Med had an average of 10 pars in the Discussion of an original article, and we have now reduced the number of paragraphs to 10. Specifically, we have moved some material to the Methods and Results sections. We stress that other reviewers asked for additional descriptions in the Discussion, which has led to an improvement of the quality of our manuscript, in our opinion.

Reviewer 4

This study applies what are in my opinion very sound methodologies to analyze the titles of prestigious, general medical journals. The paper is well-written, and its significance lays on going beyond other studies in investigating titles' form & content and the development of title content over time. To do so, they had to select a representative sample of articles over a period of 10 years. Having two (trained) raters made the methodology strong, as it was the methodology followed in the "Evaluation of title content and form" section. (I have to admit, however, that I lack the expertise to say that the statistical analyses have been performed appropriately and rigorously. So in the following I assume these have been done correctly.) By following this well-crafted methodology, and providing all the relevant data, the code to analyze it, and in detail results in the supplementary materials, the conclusions arrived at are well supported (see some comments below, though) and could be replicated by others. I do have some specific concerns or comments that I would like the authors to address:

1. I think the authors should stick to the wording "general major medical journals" instead of "highly ranked" as they don't define which "rank" that is or where it can be found or calculated.

This point has also been raised by reviewer 1, please, see above, and we have changed the wording from "high ranked" to "general major medical journals" throughout.

2. Mentioning of guidelines for authors writing the papers in the journals analyzed was not mentioned at all – even as it is mentioned in the literature they quote. I think this is important as to it may be determining why authors use a particular way to phrase the title. The reader is left to assume that no guidance was provided by the journal that could have biased title wording. I think this to be particularly important for the use or avoidance of abbreviations, dashes, and/or subtitles.

We are grateful to the reviewer for this comment. We have rewritten the Discussion. For example, we have added the sentence

> *Our findings are in line with general guidelines that declamatory titles, i.e., titles that give study results should be avoided [5]; see, e.g., instructions to authors for the Lancet.*

to the second paragraph of the Discussion.

Furthermore, we have rephrased on additional paragraph in the Discussion as follows:

> *Subtitles are now more frequently used than a decade ago. Furthermore, the mentioning of methods increased in the 10 years from 2011 to 2020. This change in*

the title may be caused by the increased use of reporting guideline, such as the CONSORT statement [7], which states that a randomized controlled trial should be identifiable as randomized in the title. The instructions for authors of all considered journals state that subtitles should be used for reporting the study design and/or authors should follow the respective reporting guidelines of their study. In fact, authors should look out a copy of the target journal and identify its preferences [8].

3. The authors recommendation "We recommend that authors study titles of articles recently published in their target journal when formulating the manuscript title" does not seem supported other than by their results implying this is what you find in them already. So, why should you follow the same? Would that make it more likely to be published? The paper's introduction makes reference to increasing citation frequency in databases, and so does at least one of the authors' previous papers, yet it's never mentioned explicitly as a possible outcome of choosing title according to the journal to submit.
Great point. The revised paragraph in the Discussion reads:

… The instructions for authors of all considered journals state that subtitles should be used for reporting the study design and/or authors should follow the respective reporting guidelines of their study. In fact, authors should look out a copy of the target journal and identify its preferences [8].

4. Regarding their recommendation "In our opinion, authors should avoid providing quantitative or semi-quantitative information in the title. In fact, since the title is a one-line summary, the conclusions could be spread out into the world without reading at least the abstract or the full text of the article." I think this argument should expand as to what the consequences are in following this behavior e.g. propagation of misinformation.
This is an important point. We have rewritten the corresponding parts in the Discussion as follows:

… Authors should thus avoid providing quantitative or semi-quantitative information in the title. In fact, since the title is a one-line summary, the conclusions could be spread out into the world without reading at least the abstract or the full text of the article. Aleixandre-Benavent and colleagues go a step further and provide recommendations what a title should contain, and how it should not be constructed [6].

... The instructions for authors of all considered journals state that subtitles should be used for reporting the study design and/or authors should follow the respective reporting guidelines of their study. In fact, authors should look out a copy of the target journal and identify its preferences [8].

5. In their statement "Another limitation of our study is that we relied on the quality of the data provided by the database of PubMed. Specifically, we may have missed some original articles in our database search. And we have previously identified a couple of errors in the database (Heßler and Ziegler, 2022)." One shouldn't expect the reader to go their paper for finding out what was wrong with those hits/articles.

We have deleted the corresponding sentence and now refer to [17]:
Cappell MS. Major bibliographic errors in PubMed: personal experience among 240 publications and proposed remediation process for errors. J Med Libr Assoc. 2016;104(1):83-4. Epub 2016/01/26. doi: 10.3163/1536-5050.104.1.015.

6. Finally, author AZ declares, in the competing interests field, that he's a "licensed Tim Albert trainer and has held several courses in the past based on Albert's concept." Please consider adding the statement that (at least some of the) Tim Albert trainings deal with advising people how to write medical papers.

Done.

P.S. There are a few typos, like missing words and letters, that need to be corrected throughout the manuscript.

We hope that we have now identified and eliminated all typos.

References

1. Heßler N, Ziegler A. Evidence-based recommendations for increasing the citation frequency of original articles. Scientometrics. 2022;127(6):3367-81. Epub May 3, 2022. doi: 10.1007/s11192-022-04378-7.
2. Kerans ME, Anne M, Sabaté S. Content and phrasing in titles of original research and review articles in 2015: range of practice in four clinical journals. Publications. 2016;4(2):11. doi: 10.3390/publications4020011.
3. Kerans ME, Marshall J, Murray A, Sabaté S. Research article title content and form in high-ranked international clinical medicine journals. English Specif Purp. 2020;60:127-39. doi: 10.1016/j.esp.2020.06.001.
4. Paiva CE, Lima JPdSN, Paiva BSR. Articles with short titles describing the results are cited more often. Clinics. 2012;67:509-13.
5. Sharp D. Kipling's guide to writing a scientific paper. Croat Med J. 2002;43(3):262-7. Epub 2002/05/30.

6. Aleixandre-Benavent R, Montalt-Resurecció V, Valderrama-Zurián JC. A descriptive study of inaccuracy in article titles on bibliometrics published in biomedical journals. Scientometrics. 2014;101(1):781-91. doi: 10.1007/s11192-014-1296-5.
7. Moher D, Hopewell S, Schulz KF, Montori V, Gotzsche PC, Devereaux PJ, et al. CONSORT 2010 explanation and elaboration: updated guidelines for reporting parallel group randomised trials. BMJ. 2010;340:c869. Epub 2010/03/25. doi: 10.1136/bmj.c869.
8. Albert T. Winning the Publications Game: The Smart Way to Write Your Paper and Get It Published. 4th ed. Boca Raton: CRC Press; 2016.
9. Fox CW, Burns CS. The relationship between manuscript title structure and success: editorial decisions and citation performance for an ecological journal. Ecol Evolut. 2015;5(10):1970-80. doi: 10.1002/ece3.1480.
10. Norman ER. Maximizing journal article citation online: readers, robots, and research visibility. Polit Policy. 2012;40(1):1-12. doi: 10.1111/j.1747-1346.2011.00342.x.
11. Haggan M. Research paper titles in literature, linguistics and science: dimensions of attraction. J Pragmat. 2004;36(2):293-317. doi: 10.1016/S0378-2166(03)00090-0.
12. Habibzadeh F, Yadollahie M. Are shorter article titles more attractive for citations? Cross-sectional study of 22 scientific journals. Croat Med J. 2010;51(2):165-70.
13. Guo F, Ma C, Shi Q, Zong Q. Succinct effect or informative effect: the relationship between title length and the number of citations. Scientometrics. 2018;116(3):1531-9. doi: 10.1007/s11192-018-2805-8.
14. Jamali HR, Nikzad M. Article title type and its relation with the number of downloads and citations. Scientometrics. 2011;88(2):653-61. doi: 10.1007/s11192-011-0412-z.
15. Bossuyt PM, Reitsma JB, Bruns DE, Gatsonis CA, Glasziou PP, Irwig L, et al. STARD 2015: an updated list of essential items for reporting diagnostic accuracy studies. BMJ. 2015;351:h5527. Epub 2015/10/30. doi: 10.1136/bmj.h5527.
16. Buter RK, van Raan AFJ. Non-alphanumeric characters in titles of scientific publications: An analysis of their occurrence and correlation with citation impact. J Inform. 2011;5(4):608-17. doi: 10.1016/j.joi.2011.05.008.
17. Cappell MS. Major bibliographic errors in PubMed: personal experience among 240 publications and proposed remediation process for errors. J Med Libr Assoc. 2016;104(1):83-4. Epub 2016/01/26. doi: 10.3163/1536-5050.104.1.015.
